# SGLT2i use is associated with reduced risks of cardiopulmonary inflammatory complications in cancer patients with diabetes: a retrospective cohort study

**DOI:** 10.3389/fcvm.2025.1657240

**Published:** 2025-09-12

**Authors:** Dan Li, Yanlin Li, Lingyu Meng, Xin Yu, Min Jiao

**Affiliations:** ^1^ Department of Medical Oncology, The First Affiliated Hospital of Xi'an Jiaotong University, Xi'an, China; ^2^ Department of Medical Oncology, The Second Affiliated Hospital of Xi'an Jiaotong University, Xi'an, China

**Keywords:** SGLT2I, type 2 diabetes mellitus, cancer, cardiovascular outcomes, respiratoryoutcomes, pneumonia, pleural effusion, pericardial effusion

## Abstract

**Background:**

Chronic low-grade inflammation constitutes a shared pathological mechanism linking type 2 diabetes mellitus (T2DM) and malignancies. While preclinical evidence suggests SGLT2 inhibitors (SGLT2i) may attenuate chronic inflammation, clinical data regarding their protective effects against multi-system inflammatory complications during anti-tumor therapy remain scarce.

**Objective:**

This study examined the association between SGLT2i use and the risk of cardiopulmonary inflammatory complications following anti-tumor therapy in cancer patients with diabetes.

**Methods:**

We conducted a retrospective, propensity score-matched cohort study at the First Affiliated Hospital of Xi'an Jiaotong University. Patients diagnosed with T2DM and cancer between March 2017 and March 2024, who survived over one year after initiating anti-tumor therapy, were included. Participants were stratified into SGLT2i users and non-users based on pre-treatment exposure. Non-SGLT2i users were matched 1:1 to users by age, sex, cancer stage, hemoglobin A1c (HbA1c), and estimated glomerular filtration rate (eGFR) levels. The primary outcome was a composite of cardiopulmonary inflammatory complications (pneumonia, pleural effusion, and pericardial effusion).

**Results:**

Among 1,183 eligible patients with T2DM and cancer, 103 received SGLT2i before anti-tumor therapy (SGLT2i group) and were matched with 103 non-SGLT2i users. Over the median follow-up period of 48 months, the SGLT2i group had a significantly lower risk of composite events (15.53% vs. 35.92%, *p* = 0.002) than the non-SGLT2i group, with reduced risks for pneumonia (9.71% vs. 22.33%, *p* = 0.030), pleural effusion (5.83% vs. 17.48%, *p* = 0.025), and pericardial effusion (2.91% vs. 10.68%, *p* = 0.030).

**Conclusion:**

In cancer patients with diabetes, pre-treatment SGLT2i use is associated with reduced risks of cardiorespiratory inflammatory complications. Robust prospective studies are warranted to confirm the role of SGLT2i in mitigating multi-system inflammatory risks in this cohort.

## 1 Introduction

Diabetes and malignancies constitute two formidable global health challenges. The clinical management of patients with both conditions represents a critical focus in metabolic oncology. Recent global epidemiological data reveal that diabetes affects approximately 828 million adults, representing a 4.2-fold increase since 1990 (1). Type 2 diabetes mellitus (T2DM) accounts for 90%–95% of these cases (2). Concurrently, cancer incidence continues to rise; the International Agency for Research on Cancer (IARC) reported approximately 20 million new cases globally in 2022, projected to reach 35 million annually by 2050 due largely to ageing populations (3). T2DM drives multiple long-term conditions (MLTCs), with cancer being a major comorbidity co-diagnosed in 15%–20% of middle-aged and older adults with diabetes at cancer diagnosis (4). Analysis of 46 million English adults demonstrates the compounding burdens: At age 50, over 30% of those with diabetes have ≥3 additional conditions. Each additional condition at the age of 50 is associated with a 4-year reduction in life expectancy, while living with these conditions for >20 years correlates with approximately 11 years earlier mortality vs. the general population (5).

At the molecular level, chronic low-grade inflammation constitutes a shared pathological mechanism linking T2DM and malignancies (6, 7). Modern anti-tumor therapy, while designed to convert immunologically “cold” tumors to “hot” tumors for enhanced efficacy, may inadvertently exacerbate inflammatory imbalance and trigger excessive immune activation (8). In cancer patients with T2DM, the convergence of hyperglycemia and therapy-induced inflammatory cascades synergistically increases adverse outcome risks. Notably, diabetes is associated with approximately 10% higher overall mortality compared to non-diabetic status (9). Consistent with recent European Society for Medical Oncology (ESMO) guidelines, patients having both T2DM and cancer experience significantly higher rates of adverse events—including cardiovascular complications, renal impairment, neuropathy, and gastrointestinal toxicity—than those with either condition alone (10).

As the global burden of diabetes and cancer escalates, advances in cancer treatment prolong survival in comorbid patients but intensify safety challenges. Conventional glucose-lowering agents, while effective for glycemic control, lack proven protective synergy in this vulnerable population. Consequently, identifying anti-diabetic drugs with dual metabolic and anti-inflammatory effects offers significant clinical promise. Sodium-glucose cotransporter-2 inhibitors (SGLT2i) are a mechanistically distinct class of oral glucose-lowering agents. SGLT2i activate AMP-activated protein kinase (AMPK) signaling, driving metabolic reprogramming that coordinates pleiotropic anti-inflammatory, antioxidant, and anti-fibrotic effects (11–14). Renal SGLT2 inhibition reduces glucose reabsorption, inducing a catabolic state of enhanced free fatty acid (FFA) utilization and ketogenesis. This metabolic rewiring attenuates adipose inflammation and oxidative stress while augmenting systemic energy availability. Ketogenesis-mediated white adipose tissue (WAT) browning and M2 macrophage repolarization synergistically improve insulin sensitivity and remodel metabolic homeostasis. SGLT2i further enhances nitric oxide (NO) bioavailability and improves antioxidant defenses via nicotinamide adenine dinucleotide phosphate (NADPH) oxidase inhibition and nuclear factor erythroid 2-related factor 2 (NRF2)/antioxidant response element (ARE) signaling activation, restoring redox balance. AMPK-silent information regulator 1 (SIRT1)-peroxisome proliferator-activated receptor γ coactivator-1α (PGC-1α) signaling upregulates autophagy and mitochondrial biogenesis. Concurrently, SGLT2i suppresses Janus kinase (JAK)/signal transducer and activator of transcription (STAT) and toll-like receptor 4 (TLR4)-myeloid differentiation primary response 88 (MyD88)-nuclear factor kappa B (NF-κB) pathways while restraining T-cell activation and inflammatory mediator release, halting inflammatory amplification. Finally, transforming growth factor-β (TGF-β)/small mothers against decapentaplegic (Smad) and mechanistic target of rapamycin complex 1 (mTORC1) inhibition curbs extracellular matrix remodeling and collagen deposition, enforcing fibroblast quiescence. Emerging translational evidence indicates SGLT2i may reduce the risk of pneumonia in diabetes (15, 16) and mitigate anthracycline-induced cardiotoxicity in cancer patients (17–19).

To bridge this gap, we conducted the first clinical evaluation of SGLT2i for multi-system inflammatory complications protection in cancer patients with diabetes. This study seeks to provide high-level evidence to optimize the management of this critical comorbidity.

## 2 Methods

### 2.1 Study design

A retrospective propensity score-matched cohort study was conducted utilizing the Biobank Database of The First Affiliated Hospital of Xi'an Jiaotong University. The study protocol was approved by the First Affiliated Hospital of Xi'an Jiaotong University (No. XJTUIAF2025LSYY-315) and complied with the Declaration of Helsinki. Informed consent was waived as this retrospective study utilized exclusively de-identified data and posed no additional risk to participants.

### 2.2 Study cohort

The single-center, prospective cohort study investigated the association between SGLT2i use and the risk of cardiopulmonary inflammatory complications following anti-tumor therapy in cancer patients with diabetes. Patients with T2DM and cancer who received anti-tumor therapy at the First Affiliated Hospital of Xi'an Jiaotong University between March 2017 and March 2024 were included. Inclusion criteria were: (1) age ≥ 18 years; (2) diagnosis of diabetes mellitus; (3) histologically or cytologically confirmed malignant tumors. Exclusion criteria were: (1) diabetes subtypes other than T2DM (e.g., secondary diabetes, gestational diabetes mellitus and type 1 diabetes mellitus); (2) presence of ≥2 primary malignancies to minimize tumor heterogeneity; (3) diabetes diagnosis after initiation of anti-tumor therapy to ensure diabetes preceded cancer; (4) pre-existing pneumonia, pleural effusion, or pericardial effusion within 30 days before anti-tumor therapy to ensure that outcomes were newly-occurring rather than the sequelae of respiratory and cardiovascular outcomes that occurred shortly before cohort entry; (5) active infections in non-cardiopulmonary systems to reduce confounding; (6) severe comorbidities: hepatic impairment (including cirrhosis or liver failure), renal impairment [estimated glomerular filtration rate (eGFR) < 30 ml/min/1.73 m^2^], autoimmune diseases, or advanced cardiac diseases (including myocardial infarction, heart valvular disease, and cardiomyopathy) as these conditions are contraindications for SGLT2i or anti-tumor therapy; (7) follow-up duration <1 year to ensure sufficient outcome observation.

The SGLT2i group included patients with any SGLT2i prescriptions before anti-tumor therapy initiation. The non-SGLT2i group received glucose-lowering agents excluding SGLT2i. The index date was defined as anti-tumor therapy initiation. Baseline data collected ≤1 year before the index date included: (1) demographics (age and sex) and body mass index (BMI); (2) cancer characteristics (cancer type/stage); (3) pre-existing cardiopulmonary diseases, underlying comorbidities, and medications; (4) imaging examinations (echocardiography, chest x-ray, and computed tomography); and (5) laboratory parameters (complete blood count with derived ratios, biochemical tests, and coagulation profiles) assessed ≤30 days prior to therapy initiation.

### 2.3 Outcome definitions

The primary outcome was a composite of cardiopulmonary inflammatory complications (pneumonia, pleural effusion, and pericardial effusion). The secondary outcomes were the individual components of the primary outcome and safety outcomes, including all-cause mortality, sepsis, neutropenic fever, urinary tract infection, and pancreatitis (detailed definitions in Supplementary Appendix). These safety outcomes were selected due to the established association of SGLT2i with infection risks in high-risk populations (17, 20–22).

### 2.4 Statistical analysis

Propensity score matching (PSM) was conducted in a 1:1 ratio to balance baseline characteristics. Propensity scores were derived via logistic regression, including these confounders: age, sex, cancer stage, hemoglobin A1c (HbA1c), and eGFR levels. Matching used a nearest-neighbor methodology with a 0.2 caliper width. Continuous variables were presented as mean ± SD and compared using independent *t*-tests (normality confirmed by the Shapiro–Wilk test). Categorical variables were presented as frequency (%) and compared via *χ*² or Fisher's exact tests. Cox proportional hazard regression was performed to evaluate associations between SGLT2i use and outcomes. Survival curves were generated using the Kaplan–Meier method and were compared by the log-rank test. Treatment-effect consistency (SGLT2i use vs. non-SGLT2i use) for the primary outcome was examined across subgroups using forest plots. All statistical analysis was performed using the R language and SPSS version 26.0, and a two-sided *p* < 0.05 was considered statistically significant.

## 3 Results

### 3.1 Baseline characteristics of study participants

**Figure 1:**
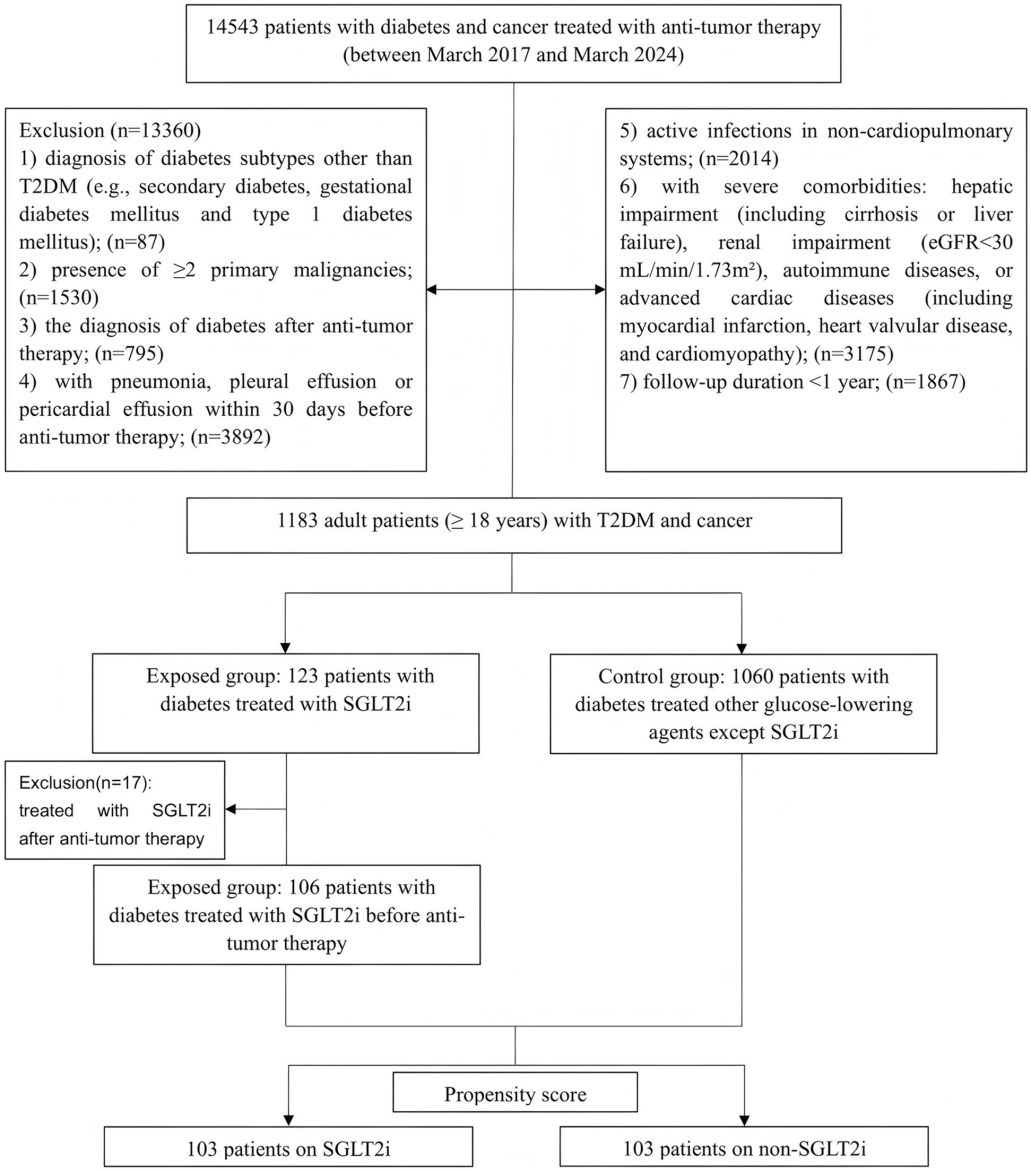
Flowchart showing patient selection. T2DM, type 2 diabetes mellitus; eGFR, estimated glomerular filtration rate; SGLT2i, Sodium-glucose cotransporter-2 inhibitors.

According to the inclusion and exclusion criteria, 1,183 patients with T2DM and cancer were enrolled. Among the 123 patients who received SGLT2i treatment, 17 patients who initiated SGLT2i after anti-tumor therapy were excluded. The remaining 1,060 patients received non-SGLT2i glucose-lowering agents. After PSM in a 1:1 ratio using predetermined variables, a final matched cohort comprising 103 patients in the SGLT2i group and 103 patients in the non-SGLT2i control group (Figure 1). Post-matching baseline characteristics are presented in Table 1. Groups were well-balanced for demographics, pre-existing cardiopulmonary diseases, and other underlying comorbidities. Cancer types and advanced-stage (IV) proportion (6.80% vs. 9.71%) were comparable. The most common cancer types in both cohorts were gastrointestinal cancers, followed by lung cancer and other malignancies. No significant differences occurred in HbA1c (7.48 ± 0.89% vs. 7.49 ± 0.87%) and eGFR (99.30 ± 18.20 vs. 99.35 ± 14.07 ml/min/1.73 m^2^) before or after matching. Similarly, inflammatory markers (including neutrophil/lymphocyte/monocyte counts and ratios) and nutritional indices (including albumin/globulin levels and ratios) showed no intergroup differences. Notably, ischemic heart disease was more prevalent in the SGLT2i group (25.24% vs. 11.65%, *p* = 0.020).

**Table 1:** Patient baseline characteristics before and after propensity score matching.

Characteristic Name	Before propensity score matching			After propensity score matching		
	Non-SGLT2i (*n* = 1,060)	SGLT2i (*n* = 106)	*P* value	Non-SGLT2i (*n* = 103)	SGLT2i (*n* = 103)	*P* value
Admission characteristics						
Age	63.65 ± 9.44	60.66 ± 9.56	0.003	60.64 ± 9.53	60.93 ± 9.38	0.825
Gender						
Female	828 (78.11)	44 (41.51)	<0.001	45 (43.69)	44 (42.72)	1.000
Male	232 (21.89)	62 (58.49)		58 (56.31)	59 (57.28)	
BMI	24.10 ± 2.88	24.67 ± 3.00	0.065	24.84 ± 3.01	24.64 ± 3.03	0.626
Systolic blood pressure	132.78 ± 17.74	127.55 ± 17.80	0.005	132.16 ± 17.62	127.30 ± 17.99	0.052
Diastolic blood pressure	78.16 ± 10.18	79.37 ± 10.23	0.247	78.84 ± 9.59	79.10 ± 10.12	0.851
Cancer stages						
I–III	975 (91.98)	96 (90.57)	0.748	96 (93.20)	93 (90.29)	0.613
IV	85 (8.02)	10 (9.43)		7 (6.80)	10 (9.71)	
Cancer types						
Head and neck	87 (8.21)	13 (12.26)	0.345	15 (14.56)	12 (11.65)	0.872
Gastrointestinal	379 (35.75)	37 (34.91)		37 (35.92)	35 (33.98)	
Hepatobiliary	77 (7.26)	5 (4.72)		7 (6.80)	5 (4.85)	
Lung carcinoma	166 (15.66)	21 (19.81)		18 (17.48)	21 (20.39)	
Others	351 (33.11)	30 (28.30)		26 (25.24)	30 (29.13)	
Laboratory data						
HbA1c	7.56 ± 0.98	7.47 ± 0.87	0.292	7.48 ± 0.89	7.49 ± 0.87	0.958
WBC (10^−9^/L)	6.62 ± 2.20	6.92 ± 2.29	0.208	6.53 ± 2.27	6.91 ± 2.31	0.229
LYMPH# (10^−9^/L)	1.58 ± 0.59	1.62 ± 0.67	0.537	1.60 ± 0.61	1.62 ± 0.67	0.860
MONO# (10^−9^/L)	0.39 ± 0.16	0.39 ± 0.17	0.812	0.41 ± 0.17	0.39 ± 0.17	0.527
NEUT# (10^−9^/L)	4.50 ± 2.26	4.74 ± 2.18	0.283	4.34 ± 2.27	4.75 ± 2.20	0.196
EO# (10^−9^/L)	0.13 ± 0.14	0.12 ± 0.12	0.622	0.15 ± 0.17	0.12 ± 0.12	0.249
PLT (10^−9^/L)	217.74 ± 68.73	228.20 ± 73.90	0.165	207.37 ± 76.28	224.47 ± 70.03	0.095
FIB	3.45 ± 0.91	3.48 ± 0.92	0.708	3.23 ± 0.74	3.50 ± 0.92	0.025
eGFR	101.40 ± 18.21	98.74 ± 14.73	0.086	99.30 ± 18.20	99.35 ± 14.07	0.985
LDH	205.48 ± 61.22	210.15 ± 61.06	0.454	202.65 ± 44.06	210.48 ± 61.90	0.297
GLO	26.48 ± 4.79	26.88 ± 4.25	0.369	26.61 ± 4.72	26.89 ± 4.26	0.660
ALB	40.45 ± 4.59	40.46 ± 4.35	0.981	40.33 ± 4.53	40.39 ± 4.39	0.923
NLR	3.61 ± 3.98	3.73 ± 3.13	0.721	3.41 ± 3.34	3.73 ± 3.15	0.474
PLR	156.96 ± 84.54	164.77 ± 89.21	0.390	148.14 ± 90.97	162.15 ± 85.95	0.257
LMR	4.54 ± 2.16	4.72 ± 2.83	0.526	4.44 ± 2.09	4.75 ± 2.86	0.363
PNI	48.35 ± 5.78	48.57 ± 5.49	0.695	48.35 ± 5.20	48.49 ± 5.50	0.853
AGR	1.57 ± 0.31	1.54 ± 0.28	0.270	1.57 ± 0.36	1.54 ± 0.28	0.491
SII	783.08 ± 896.11	847.60 ± 774.91	0.422	684.03 ± 673.46	832.12 ± 754.36	0.139
Comorbidities						
Hypertension	583 (55.00)	59 (55.66)	0.978	53 (51.46)	56 (54.37)	0.780
Ischemic heart diseases	142 (13.40)	28 (26.42)	0.001	12 (11.65)	26 (25.24)	0.020
Cerebrovascular disease	91 (8.58)	13 (12.26)	0.276	5 (4.85)	13 (12.62)	0.084
Hyperlipidemia	14 (1.32)	6 (5.66)	0.007	1 (0.97)	6 (5.83)	0.119
CKD	5 (0.47)	1 (0.94)	0.436	2 (1.94)	1 (0.97)	1.000
Other endocrine diseases	11 (1.04)	5 (4.72)	0.011	0 (0.00)	5 (4.85)	0.059
COPD/Chronic bronchitis	8 (0.75)	1 (0.94)	0.577	3 (2.91)	1 (0.97)	0.621
Concomitant medications						
ACEI/ARB	200 (18.87)	27 (25.47)	0.131	22 (21.36)	25 (24.27)	0.740
Diuretics	47 (4.43)	6 (5.66)	0.621	6 (5.83)	6 (5.83)	1.000
Statin	80 (7.55)	18 (16.98)	0.002	12 (11.65)	17 (16.50)	0.423
Aspirin	116 (10.94)	16 (15.09)	0.260	16 (15.53)	15 (14.56)	1.000
Clopidogrel/Ticagrelor	30 (2.83)	7 (6.60)	0.072	3 (2.91)	6 (5.83)	0.498
Beta-blockers	82 (7.74)	15 (14.15)	0.036	8 (7.77)	14 (13.59)	0.259
Calcium channel blockers	304 (28.68)	31 (29.25)	0.992	33 (32.04)	28 (27.18)	0.542

SGLT2i, sodium-glucose cotransporter 2 inhibitors; BMI, body mass index; HbA1c, glycated hemoglobin; WBC, white blood cell; LYMPH#, lymphocyte count; MONO#, monocyte count; NEUT#, neutrophil count; EO#, eosinophil; PLT, platelet; FIB, fibrinogen; eGFR, estimated glomerular filtration rate; LDH, lactate dehydrogenase; GLO, globulin; ALB, albumin; NLR, neutrophil to lymphocyte ratio; PLR, platelet to lymphocyte ratio; LMR, lymphocyte to monocyte ratio; PNI, prognostic nutritional index; SII, systemic immune-inflammatory index; CKD, chronic kidney disease; COPD, chronic obstructive pulmonary disease; ACEI/ARB, angiotensin-converting enzyme inhibitors/angiotensin receptor blockers. Measurement data are described as mean ± SD (standard deviation). Enumeration data are presented as count (percent).

### 3.2 Cardiopulmonary outcomes

**Figure 2:**
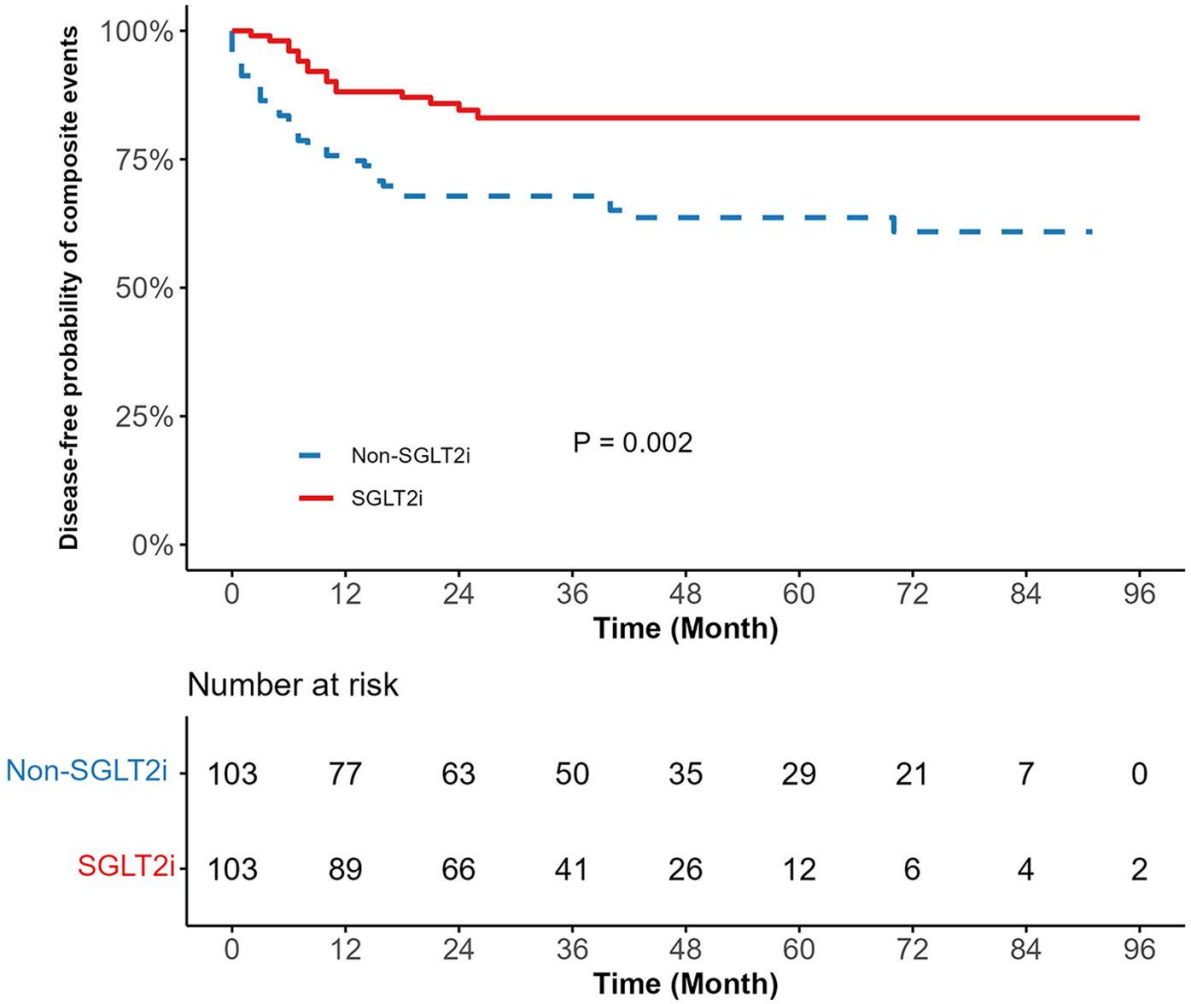
Kaplan meier survival curve comparing composite events between SGLT2i and non-SGLT2i. SGLT2i, sodium-glucose cotransporter-2 inhibitors. The blue line represents the non-SGLT2i group, and the red line represents the SGLT2i group. The *p*-value is the result of the log-rank test (*p* = 0.002).

Kaplan–Meier survival curves depicting the cardiopulmonary outcomes are shown in Figure 2 and Supplementary Figures S1–S3. The SGLT2i group exhibited a significantly lower risk of composite events (15.53% vs. 35.92%, *p* = 0.002) than the non-SGLT2i group, with reduced risks for pneumonia (9.71% vs. 22.33%, *p* = 0.030), pleural effusion (5.83% vs. 17.48%, *p* = 0.025), and pericardial effusion (2.91% vs. 10.68%, *p* = 0.030). Supplementary Table S1 indicates longer mean event-free survival time among SGLT2i users for composite events (81.77 months vs. 61.16 months), pneumonia (87.14 months vs. 72.81 months), pleural effusion (90.72 months vs. 76.51 months), and pericardial effusion (93.40 months vs. 82.33 months). Over the median follow-up period of 48 months, 16 composite events occurred in the SGLT2i group compared to 37 events in the non-SGLT2i group. A univariate Cox proportional hazards analysis revealed an approximately 60% lower composite events risk in SGLT2i users (HR = 0.40, 95% CI: 0.23–0.73, *p* = 0.003). Similarly reduced risks were observed for pneumonia (HR = 0.45, 95% CI: 0.21–0.95, *p* = 0.036), pleural effusion (HR = 0.36, 95% CI: 0.14–0.92, *p* = 0.033), and pericardial effusion (HR = 0.27, 95% CI: 0.08–0.97, *p* = 0.045) (Table 2).

**Table 2:** Cox proportional hazard analysis for the association between the use of SGLT2i versus non-SGLT2i on patient outcomes.

Outcome Type	Non-SGLT2i (*N* = 103)	SGLT2i (*N* = 103)	Hazard Ratio (95% CI)	*P* Value
Cardiopulmonary outcomes				
Composite events	37	16	0.40 (0.23–0.73)	0.003
Pneumonia	23	10	0.45 (0.21–0.95)	0.036
Pleural effusion	18	6	0.36 (0.14–0.92)	0.033
Pericardial effusion	11	3	0.27 (0.08–0.97)	0.045
Safety outcomes				
All-cause mortality	17	9	0.72 (0.32–1.65)	0.439
Sepsis	12	3	0.25 (0.07–0.90)	0.034
Neutropenic fever	3	1	0.38 (0.04–3.68)	0.404
Urinary tract infection	4	2	0.56 (0.10–3.07)	0.505
Pancreatitis	4	2	0.57 (0.10–3.11)	0.515

SGLT2i, sodium-glucose cotransporter 2 inhibitors; HR, hazard ratio; CI, confidence intervals.

### 3.3 Safety outcomes

During follow-up, there were 9 and 17 deaths in the SGLT2i and non-SGLT2i groups, respectively. The SGLT2i group exhibited numerically lower all-cause mortality (8.74% vs. 16.50%, *p* = 0.437) compared to the non-SGLT2i group (Table 2 and Supplementary Table S1). Notably, sepsis incidence was significantly lower in the SGLT2i group (HR = 0.25, 95% CI: 0.07–0.90, *p* = 0.034). Kaplan–Meier curves for sepsis are presented in Supplementary Figure S4. No significant differences emerged in neutropenic fever, urinary tract infection, and pancreatitis risks between groups (Table 2).

### 3.4 Sensitivity analysis

**Figure 3:**
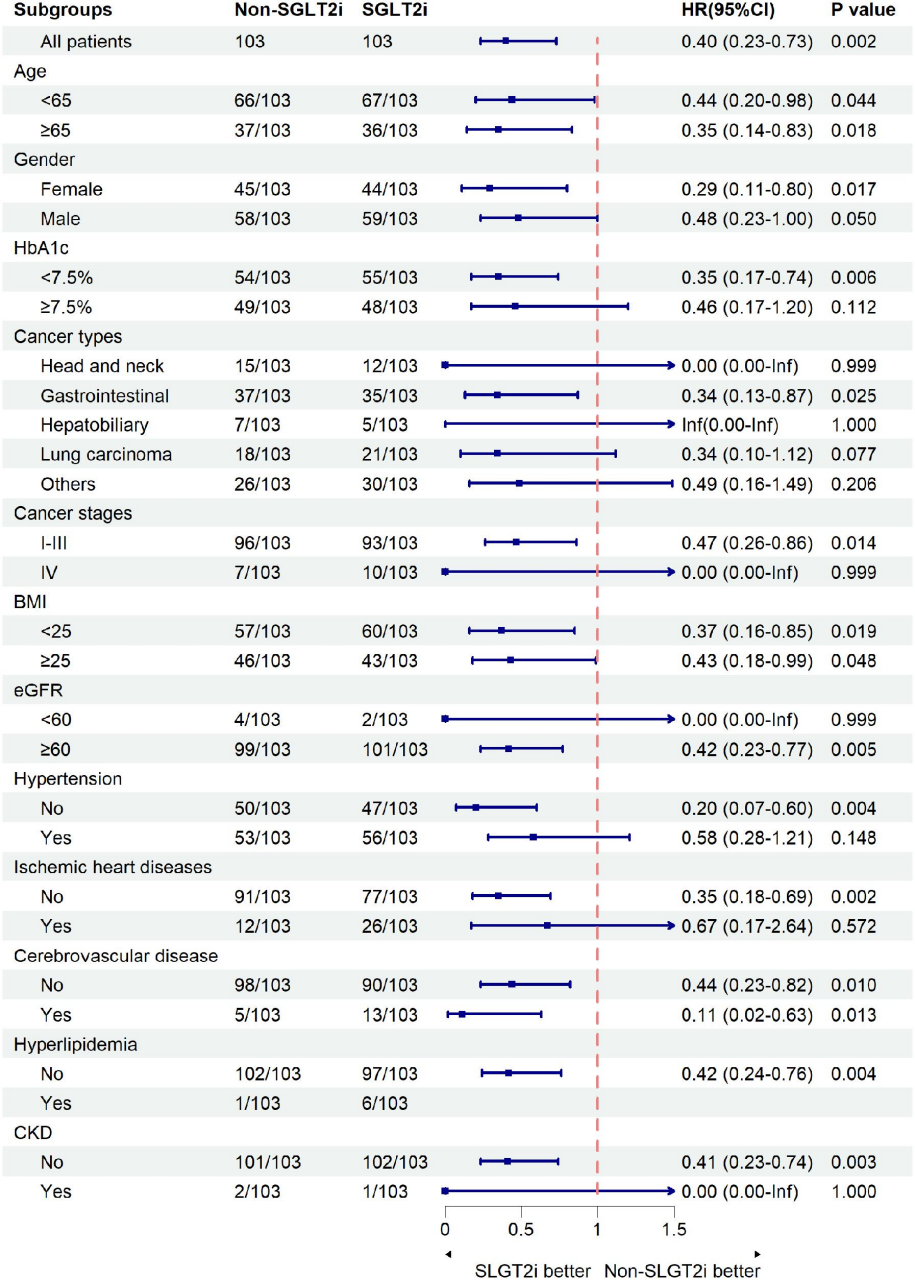
Composite events in subgroups. The composite events were defined as pneumonia, pleural effusion, or pericardial effusion. SGLT2i, sodium-glucose cotransporter-2 inhibitors; HbA1c, glycated hemoglobin; BMI, body mass index; eGFR, estimated glomerular filtration rate; CKD, chronic kidney disease. The size of the boxes is proportional to the number of patients in the subgroup, and arrows on the confidence interval bars indicate that the upper or lower boundary of the confidence interval is off the scale.

Subgroup analysis demonstrated consistent protective effects of SGLT2i against composite events across most predefined subgroups (Figure 3). Significant risk reductions occurred in: age <65 years (HR = 0.44, 95% CI: 0.20–0.98, *p* = 0.044) and ≥65 years (HR = 0.35, 95% CI: 0.14–0.83, *p* = 0.018), HbA1c < 7.5% (HR = 0.35, 95% CI: 0.17–0.74, *p* = 0.006), eGFR ≥60 ml/min/1.73 m^2^ (HR = 0.42, 95% CI 0.23–0.77, *p* = 0.005), non-hypertensive patients(HR = 0.20, 95% CI: 0.07–0.60, *p* = 0.004), patients without ischemic heart disease (HR = 0.35, 95% CI: 0.18–0.69, *p* = 0.002), patients without chronic kidney disease (HR = 0.41, 95% CI: 0.23–0.74, *p* = 0.003), and cerebrovascular disease subgroup (HR = 0.11, 95% CI: 0.02–0.63, *p* = 0.013). Point estimates favored the SGLT2i in all subgroups except for hyperlipidemia, which had only one control patient. Benefit magnitude was greater in females (HR = 0.29) than in males (HR = 0.48) and in early-stage (I-III) cancer (HR = 0.47) vs. stage IV disease (HR not estimable).

## 4 Discussion

This propensity score-matched cohort study provides the first clinical evidence that pre-treatment SGLT2i use is associated with the risk of reduced cardiopulmonary inflammatory complications in cancer patients with diabetes. Our principal findings are: (1) Patients who received SGLT2i had lower risks of systemic inflammatory complications than those who received non-SGLT2i glucose-lowering agents; (2) SGLT2i appear to be safe in this group of patients at high risk, correlating with reduced sepsis incidence. While the reduction in overall mortality did not reach statistical significance, this trend aligns with most existing literature and may achieve significance in larger cohorts.

SGLT2i reduces blood glucose through SGLT2 receptor inhibition in renal proximal tubules. Beyond glucose-lowering effects, SGLT2i confer cardiorenal protection in diabetic and non-diabetic populations through pleiotropic mechanisms—including suppression of inflammatory pathways, attenuation of fibrotic signaling, and mitigation of oxidative stress (23, 24). Our study specifically examines comorbidity management in cancer patients with diabetes. Supporting this focus, observational studies suggest SGLT2i may mitigate adverse cardiac events in T2DM patients with cancer receiving anthracycline-based therapies (18, 19, 25). Consistently, we observed SGLT2i use was associated with a significantly lower incidence of pericardial effusion (2.91% vs. 10.68%, *p* = 0.030). Given the risks of diabetes-associated infection (26), SGLT2i may reduce adverse respiratory events through dual mechanisms: inherent anti-inflammatory effects and osmotic diuresis. Large retrospective cohort studies support a reduced risk of respiratory events in T2DM patients (27–29). In cancer patients with diabetes, chronic low-grade inflammation within the tumor microenvironment (TME) and anti-tumor therapy-induced hyperinflammation may exacerbate respiratory complications. Notably, SGLT2i use was associated with significantly reduced incidences of pneumonia (9.71% vs. 22.33%, *p* = 0.030) and pleural effusion (5.83% vs. 17.48%, *p* = 0.025).

Diabetes-associated chronic low-grade inflammation drives cellular and tissue damage, DNA damage, and mutation, ultimately promoting tumorigenesis, invasion, and metastasis (30). Conversely, tumor growth induces tissue remodeling, fostering an immunosuppressive, inflammatory TME (31). Anti-tumor therapy, while aiming to reverse immunosuppression through immunogenic cell death (ICD) and the release of pathogen-associated molecular patterns (PAMPs) or damage-associated molecular patterns (DAMPs) that engage pattern recognition receptors (PRRs), may paradoxically trigger hyperinflammation (32). Preclinical models demonstrate SGLT2i's benefits for the respiratory and cardiovascular systems in diabetic or tumor-bearing mice. Dapagliflozin reduced airway surface liquid (ASL) glucose and bacterial load in infected with Pseudomonas aeruginosa (33). Both dapagliflozin and empagliflozin improved cardiac function through anti-inflammatory and antioxidant mechanisms (34, 35). Furthermore, SGLT2i-induced weight loss, achieved through reduced insulin resistance (36, 37), attenuates obesity-driven chronic inflammation—a core pathophysiological feature of T2DM (38). A large meta-analysis further confirms SGLT2i-induced reductions in systemic inflammatory mediators (39).

Recent meta-analyses indicate that SGLT2i use does not increase overall cancer risk (40, 41). Conversely, preclinical studies suggest potential anti-tumor mechanisms operating both dependently and independently of SGLT2 inhibition, including impeding tumor glucose uptake, promoting apoptosis, altering oncogenic signaling, inducing cell cycle arrest, inhibiting angiogenesis, enhancing anti-tumor immunity, and attenuating chronic inflammation (42). Retrospective evidence associates SGLT2i with reduced risks of lung, prostate, and gastrointestinal cancers (43–45). Notably, one cohort study reported an approximately 46% lower all-cause mortality in non-small cell lung cancer (NSCLC) patients vs. non-users (44). Critically, randomized clinical trials (RCTs) confirm SGLT2i promote weight loss without compromising muscle mass or strength (46), and generally does not increase risks of hypovolemia, renal impairment/failure, fracture, diabetic ketoacidosis, amputation, or severe hypoglycemia (Exceptions: dapagliflozin [urinary tract infection]; ertugliflozin/ipragliflozin [genital infection]) (47, 48). Our safety data corroborate this profile. SGLT2i use was associated with an approximately 75% lower incidence of sepsis (2.91% vs. 11.65%, *p* = 0.021), consistent with the literature (15, 16, 49, 50), and no increased risk of neutropenic fever, urinary tract infection, or pancreatitis. Although mortality reduction remained non-significant (8.74% vs. 16.50%, *p* = 0.437), this trend warrants validation in larger prospective multicenter studies.

Subgroup analyses further indicate SGLT2i confer anti-inflammatory benefits beyond glycemic control in cancer patients with diabetes, particularly those without pre-existing hypertension, cardiovascular/cerebrovascular diseases, or renal impairment. Notably, patients with gastrointestinal cancers exhibited a striking 66% reduction in risk, a finding requiring prospective validation. Study limitations include: (1) limited precision in smaller subgroups (e.g., hepatobiliary cancers), precluding reliable hazard ratio estimation—addressed by future studies using larger databases; (2) enhanced protection in females suggesting estrogen-SGLT2i interplay, a hypothesis needing preclinical investigation; (3) underrepresentation of stage IV cancer patients, limiting conclusions and necessitating broader-inclusion follow-up studies.

### 4.1 Limitations and future research directions

Inherent to its retrospective design, this study has limitations. Despite employing PSM to minimize baseline differences and potential confounders and incorporating critical variables such as underlying disease severity and eGFR, residual confounding may affect the association between SGLT2i use and inflammatory/safety outcomes. Furthermore, all data analyzed in the study were retrospectively extracted from the electronic medical record system, which may potentially introduce biases from mis-coding or mis-diagnosis of outcomes, but such biases are likely to impact both the SGLT2i and non-SGLT2i groups. Owing to the limited sample size, we could not confidently detect a mortality benefit from SGLT2i nor could we conduct head-to-head comparisons against other glucose-lowering medications (e.g., glucagon-like peptide-1 receptor agonists or dipeptidyl peptidase 4 inhibitors). Detailed subgroup analyses—by concomitant anti-tumor therapies, specific SGLT2i, dosages, or treatment durations—were likewise underpowered. The generalizability of our results is also tempered by the study's stringent inclusion criteria, a necessary trade-off that future studies in broader populations should address. It is also important to note that our findings, confined to a T2DM population, may not extend to individuals without diabetes.

## 5 Conclusion

In this propensity score-matched cohort study, pre-treatment SGLT2i use in cancer patients with diabetes was associated with lower risks of cardiopulmonary inflammatory complications without raising any new safety concerns.

## Data Availability

The raw data supporting the conclusions of this article will be made available by the authors, without undue reservation.
