# Supplementary material for: SGLT2i use is associated with reduced risks of cardiopulmonary inflammatory complications in cancer patients with diabetes: a retrospective cohort study
Source: Front Cardiovasc Med. 2025 Sep 12;12:1657240. doi: 10.3389/fcvm.2025.1657240 (PMC12463981; doi:10.3389/fcvm.2025.1657240)
Supplement: Supplementary file 1 [file Datasheet1.pdf]

Supplementary Material

1 Supplementary Data

Supplementary Material should be uploaded separately on submission. Please include any supplementary data, figures and/or tables.

Supplementary material is not typeset so please ensure that all information is clearly presented, the appropriate caption is included in the file and not in the manuscript, and that the style conforms to the rest of the article.

2 Supplementary Figures and Tables

2.1 Supplementary Figures

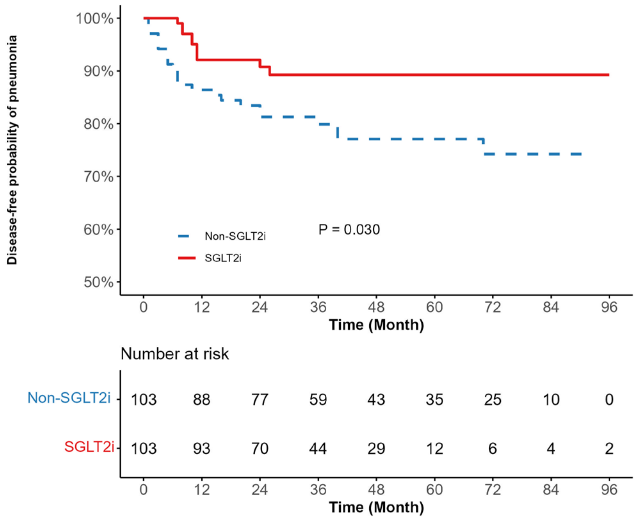

Supplementary Figure 1. Kaplan Meier Survival Curve comparing pneumonia between SGLT2i and non-SGLT2i. Abbreviations: SGLT2i, Sodium-glucose cotransporter-2 inhibitors. The blue line

represents the non-SGLT2i group, and the red line represents the SGLT2i group. The P-value is the result of the log-rank test (P = 0.030).

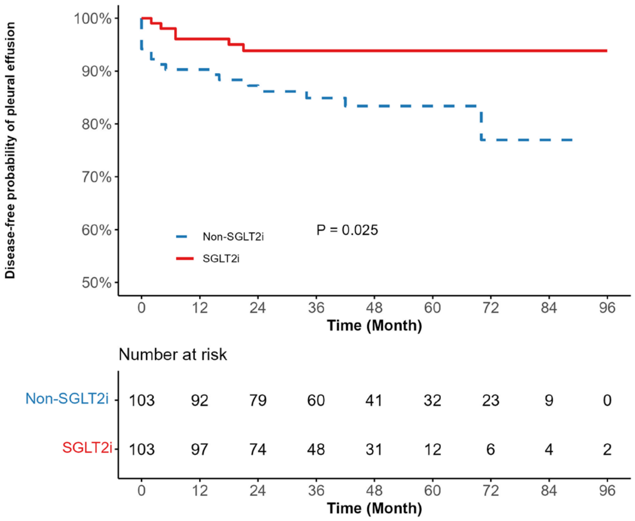

Supplementary Figure 2. Kaplan Meier Survival Curve comparing pleural effusion between SGLT2i and non-SGLT2i. Abbreviations: SGLT2i, Sodium-glucose cotransporter-2 inhibitors. The blue line represents the non-SGLT2i group, and the red line represents the SGLT2i group. The P-value is the result of the log-rank test (P = 0.025).

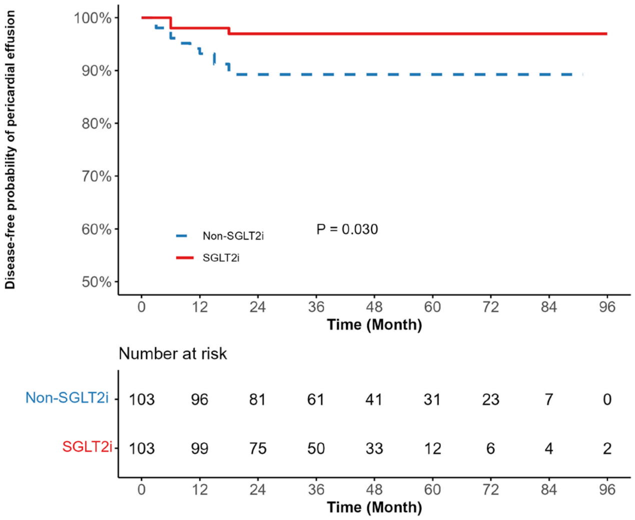

Supplementary Figure 3. Kaplan Meier Survival Curve comparing pericardial effusion between SGLT2i and non-SGLT2i. Abbreviations: SGLT2i, Sodium-glucose cotransporter-2 inhibitors. The

blue line represents the non-SGLT2i group, and the red line represents the SGLT2i group. The P-value is the result of the log-rank test (P = 0.030).

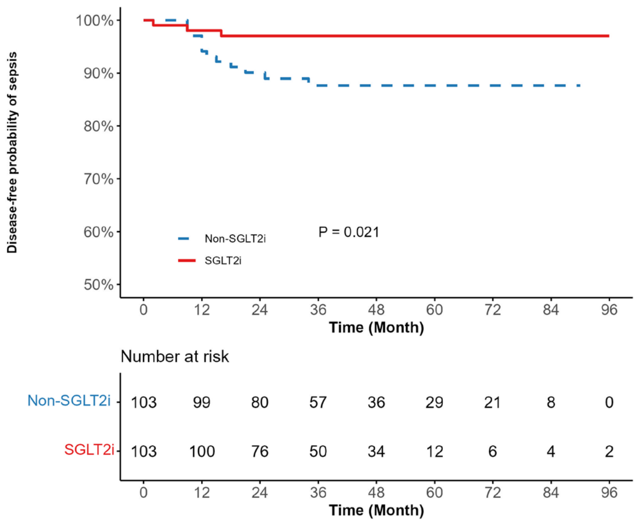

Supplementary Figure 4. Kaplan Meier Survival Curve comparing sepsis between SGLT2i and non-SGLT2i. Abbreviations: SGLT2i, Sodium-glucose cotransporter-2 inhibitors. The blue line represents the non-SGLT2i group, and the red line represents the SGLT2i group. The P-value is the result of the log-rank test (P = 0.021).

2.2 Supplementary Tables

Supplemental Appendix

**Disease**	**Definition**
Pneumonitis	A disorder characterized by inflammation focally or diffusely affecting the lung parenchyma.
Pleural effusion	A disorder characterized by an increase in amounts of fluid within the pleural cavity. Symptoms include shortness of breath, cough and marked chest discomfort.
Pericardial effusion	A disorder characterized by fluid collection within the pericardial sac, usually due to inflammation.
Sepsis	A disorder characterized by the presence of pathogenic microorganisms in the bloodstream that cause a rapidly progressing systemic reaction that may lead to shock.
Neutropenic fever	A disorder characterized by a single oral temperature measurement of ≥38.3°C (101°F) or a temperature of ≥38.0°C

	(100.4°F) sustained over 1 hour in the setting of an absolute neutrophil count (ANC) <500 cells/μL, or an ANC expected to fall to <500 cells/μL within 48 hours.
Urinary tract infection	A disorder characterized by an infectious process involving the urinary tract, most commonly the bladder and the urethra.
Pancreatitis	A disorder characterized by inflammation of the pancreas with no documented pancreas infection.

Table S1 Log-rank survival analysis for the use of SGLT2i versus non-SGLT2i

**Outcome Type**	**Non-SGLT2i (N=103)**	**SGLT2i (N=103)**	χ^2	**P Value (Log-rank)**
**Cardiopulmonary outcomes**				
Composite events	61.16 (53.44,68.89)	81.77 (75.37,88.17)	9.859	0.002
Pneumonia	72.81 (66.24,79.37)	87.14 (81.92,92.36)	4.696	0.030
Pleural effusion	76.51 (70.42,82.59)	90.72 (86.62,94.82)	5.004	0.025
Pericardial effusion	82.33 (77.47,87.18)	93.40 (90.49,96.30)	4.682	0.030
**Safety outcomes**				
Overall mortality	79.86 (74.81,84.91)	85.36 (78.03,92.69)	0.605	0.437
Sepsis	80.91 (76.09,85.74)	93.43 (90.55,96.30)	5.291	0.021
Neutropenic fever	88.76 (86.26,91.26)	95.16 (93.52,96.80)	0.750	0.386
Urinary tract infection	87.92 (84.96,90.88)	94.27 (91.89,96.65)	0.458	0.499
Pancreatitis	88.06 (85.24,90.88)	94.36 (92.11,96.61)	0.436	0.509

Abbreviations: SGLT2i, sodium-glucose cotransporter 2 inhibitors
